# Critical Analysis and Cross-Comparison Between English and Chinese Websites Providing Online Medical Information for Patients With Adenoid Hypertrophy: Cross-sectional Study

**DOI:** 10.2196/44010

**Published:** 2023-04-24

**Authors:** Zheng Jiang, Xin Yang, Fei Chen, Jun Liu

**Affiliations:** 1 Department of Otolaryngology, Head and Neck Surgery West China Hospital Sichuan University Chengdu China

**Keywords:** adenoid hypertrophy, website quality, critical analysis, English and Chinese, English, Chinese, patient, internet, online, decisions, medical issues, airway obstruction, airway, accessibility, quality

## Abstract

**Background:**

In the information era, patients can easily be misled by inaccurate internet content, thus making not well-informed decisions about medical issues. Adenoid hypertrophy, one of the most common causes of chronic upper airway obstruction in children and adolescents, may lead to serious complications, including sleep apnea and craniofacial change. There have been no critical studies about the quality of websites on adenoid hypertrophy, posing a challenge for users without a medical background to determine which website offers more reliable information. Moreover, the blockage of access to internet search tools such as Google, Yahoo, and others has created an isolated internet environment for the enormous user population in mainland China. Differences in internet legislation, the commercial environment, and culture are also likely to result in varied quality of online health information inside and outside mainland China. To date, no study has compared the quality difference between mainland Chinese and English websites.

**Objective:**

The aims of this study were to (1) analyze the quality of websites about adenoid hypertrophy accessible by patients, (2) investigate the quality differences between Chinese and English websites, (3) determine which type of website (eg, government-sponsored, health care provider) is more reliable in terms of medical information, and (4) determine whether the blockage of foreign websites is hindering users’ accessibility to better-quality websites in mainland China.

**Methods:**

The first 100 websites (excluding advertisements) displayed on the top three search engines worldwide and in mainland China for the key search term “enlarged adenoids” were collected as the data source. The websites were evaluated based on accessibility, accountability, interactivity, structure, and content quality (accuracy, content coverage, and objectivity). Cohen κ was calculated, and one-way ANOVA and the Kruskal-Wallis test were performed to compare the results between groups and subgroups.

**Results:**

The mean score for the content quality of English websites was significantly higher than that of Chinese websites (6.16 vs 4.94, *P*=.03 for Google, Bing, and Yahoo; 6.16 vs 4.16, *P*<.001 for Baidu, Sougou, and Bing China). Chinese users who are not influenced by the Internet Censorship System are more likely to access higher-quality online medical information (4.94 vs 4.16, *P*=.02). In within-group Student-Newman-Keuls *q* posthoc analysis, professional organization and government-sponsored websites were generally of better quality than other websites for both Chinese and English websites (*P*<.05).

**Conclusions:**

Generally, the English websites on adenoid hypertrophy are of better quality than Chinese websites; thus, Chinese users residing outside of the Chinese mainland are less influenced by inaccurate online medical information.

## Introduction

Adenoid hypertrophy, as the most common cause of chronic upper airway obstruction in children, can be worrisome to parents. Adenoid hypertrophy can cause mouth breathing, nasal congestion, hyponasal speech, snoring, and obstructive sleep apnea, and can potentially lead to serious complications in the long-term, such as growth retardation, cardiovascular abnormality, and craniofacial change [1]. A recent meta-analysis revealed an overall prevalence of adenoid hypertrophy among children of 34.46% [1], suggesting that nearly 1 in 3 children has experienced adenoid hypertrophy. The treatment of choice is based on the etiology and severity of symptoms. For patients with infectious adenoid hypertrophy, antibiotics such as amoxicillin or azithromycin are recommended. Nasal steroids and some nasal decongestants can offer symptom relief, but the evidence is mixed [2,3]. For patients with recurrent or persistent obstructive or infectious symptoms, adenoidectomy with or without tonsillectomy can be effective [4]. Any delayed treatment can potentially lead to irreversible complications.

A key to improving children’s health outcomes is to help their parents make well-informed decisions. However, under the information era, learning and making decisions about a medical issue can be both easy and difficult. The popularization of convenient access to the internet makes the process of gaining health information simple, and people are now used to searching online for medical issues. Taking Canada as an example of English users, 70% of Canadians reported having searched medical or health-related information on the internet [5]. A survey performed in China revealed that 79% of adults had used the internet as their source of medical knowledge [6]. A systematic review showed that children and adolescents, who are generally good at using technology, search online for specific health-related information as the internet is their most frequently used information source [7]. Although the internet can provide infinite information from various sources conveniently, it may be hard for people to discern the quality of the information obtained. A systematic review concluded that approximately 70% of studies found the quality of health information to be problematic on the internet [8]. Therefore, parents and their children as internet users without proper medical knowledge can be misled by inaccurate internet contents, posing a challenge to make well-informed decisions.

For various reasons, the Chinese government has blocked the domestic access of several foreign internet tools such as Google Search, Yahoo, and others [9]. This policy has created an isolated internet environment for the enormous domestic user population from mainland China, which has developed its own search engines, social media platforms, and internet content [10]. For example, the market of for-profit online health communities in China is very prosperous due to the very uneven distribution of medical resources, which indeed help people maintain a healthy lifestyle and reduce the burden on the health care system [11]. The primary social media platform WeChat also plays an important role in providing medical information, as approximately 63.26% of users use WeChat to obtain health information in China [12]. Despite the existence of various health information providers, the Chinese people’s priority choice in seeking medical information remains online websites [6]. The differences in internet legislation, the commercial environment, and distribution of medical resources are likely to result in varied quality of online health information inside and outside mainland China.

To date, no study has compared the quality of online health information between the Chinese mainland and the rest of the world; thus, it remains unclear whether internet isolation is limiting Chinese people from obtaining high-quality online health information. In addition, to our knowledge, there have been no critical studies assessing the quality of online content on adenoid hypertrophy, in English or Chinese. Therefore, the aims of this study were to (1) address this knowledge gap by assessing the quality of online content on adenoid hypertrophy and (2) compare the quality of online health information between Chinese and English websites.

## Methods

### Search Strategy

This was a cross-sectional study assessing online patient educational content on adenoid hypertrophy. We conducted three searches in the Chinese mainland and Hong Kong Special Administrative Region (SAR) on August 1, 2022, and formed three groups: group 1 included English websites searched with Google, Yahoo, and Bing (the three most frequently used search engines worldwide [13]); group 2 included Chinese websites searched with Google, Yahoo, and Bing (representing the Chinese users residing outside of mainland China); and group 3 included Chinese websites searched with Baidu, Sougou, and Bing China (as the most frequently used search engines in China [14]). When searching with Google, Yahoo, and Bing, a Hong Kong SAR IP address was used and when searching with Baidu, Sougou, and Bing China, a Chinese mainland IP address was used.

“Enlarged adenoids” and its Chinese translation were used as the search terms since this term is more frequent on Google Trends than “adenoid hypertrophy.” We collected the first 100 websites from each search engine, excluding advertisements, to ensure inclusivity, since the majority of internet searchers will not go beyond the first page or top 10 search results for both general and health-related information [15]. Inclusion and exclusion criteria are provided in Textbox 1.

**Inclusion and exclusion criteria.**

**Inclusion criteria**

- Websites intended for the purpose of patient education that provide information related to adenoid hypertrophy
- Websites accessible without subscription or registration

**Exclusion criteria**

- Sites exclusively dedicated to fundraising, pharmaceuticals, and advertising treatments, and not containing any specific adenoid information for patients
- Specialized websites designed for health professionals
- Webpages solely based on a mailing list or discussion board, blogs not written by medical professionals
- Decision tools
- Websites with no unique information, merely providing links to other sites, other published materials (journal or PDF), search engines, or resource directories

### Site Traffic

The site traffic of English websites was assessed using SEMrush, whereas that of the Chinese websites was assessed using a search engine optimization (SEO) tool (chinaz) since SEMrush does not contain data of site views from mainland China.

### Assessment

We adapted the assessment tool proposed by Ingledew [16] for our adenoid hypertrophy assessment. Accuracy was graded by comparing the webpage information with reliable resources such as UptoDate, Pan-American Clinical Practice Guideline for Medical Management of Acute Tonsillitis and Adenoids Hypertrophy, and National Center for Biotechnology Information (NCBI) bookshelf. The websites were further categorized into five types: (1) government-sponsored, (2) hospital/health care provider, (3) professional organization, (4) industry-sponsored, and (5) other commercial websites.

### Assessment Tool Evaluation and Rater Training

Two raters (ZJ and XY) independently applied the tool to 20 random adenoid hypertrophy websites and Cohen κ was calculated [17]; fields with an interrater correlation coefficient <0.7 were modified to reduce ambiguity and consensus was reached after reviewing the discrepancies. The final website assessment was performed by ZJ alone from August 2 to August 27, 2022. The assessment tool contained six parts: accessibility (total score=1), accountability (total score=15), interactivity (total score=5), structure and organization (total score=5), accuracy (total score=5, used for weighting the medical content only), and medical content (total score=15). The detailed assessment tool design is provided in Multimedia Appendix 1.

### Statistical Analysis

Categorical data are presented as percentages and numerical data are presented as mean (SD). The final medical content score was weighted based on the accuracy score (0%-0%, 1%-20%, 2%-40%, 3%-60%, 4%-80%, 5%-100%). The following formulas were used for calculating the website score:

Website design score (without medical content score)=Accessibility+Accountability+Interactivity+Structure and organization

Medical content quality score=Content×Accuracy×0.2 (weighted based on the accuracy score for a total score of 5)

Website total quality score=Website design score+Medical content quality score One-way ANOVA followed by the Student-Newman-Keuls *q* posthoc test or the Kruskal-Wallis *H* test followed by Mann-Whitney *U* tests were used to compare the score differences among the three groups and in subgroup analysis. The Pearson correlation coefficient (*r*) was used to assess the correlation between website score and site traffic. All statistical analyses were performed with SPSS software (version 23.0). The significance level was set at *P*<.05.

### Ethical Considerations

Institutional review board approval was not required for this study since all information was freely available online and the study did not include any patient data. All the websites included for this analysis are open access.

## Results

### Basic Descriptive Data of Included Websites

**Figure 1:**
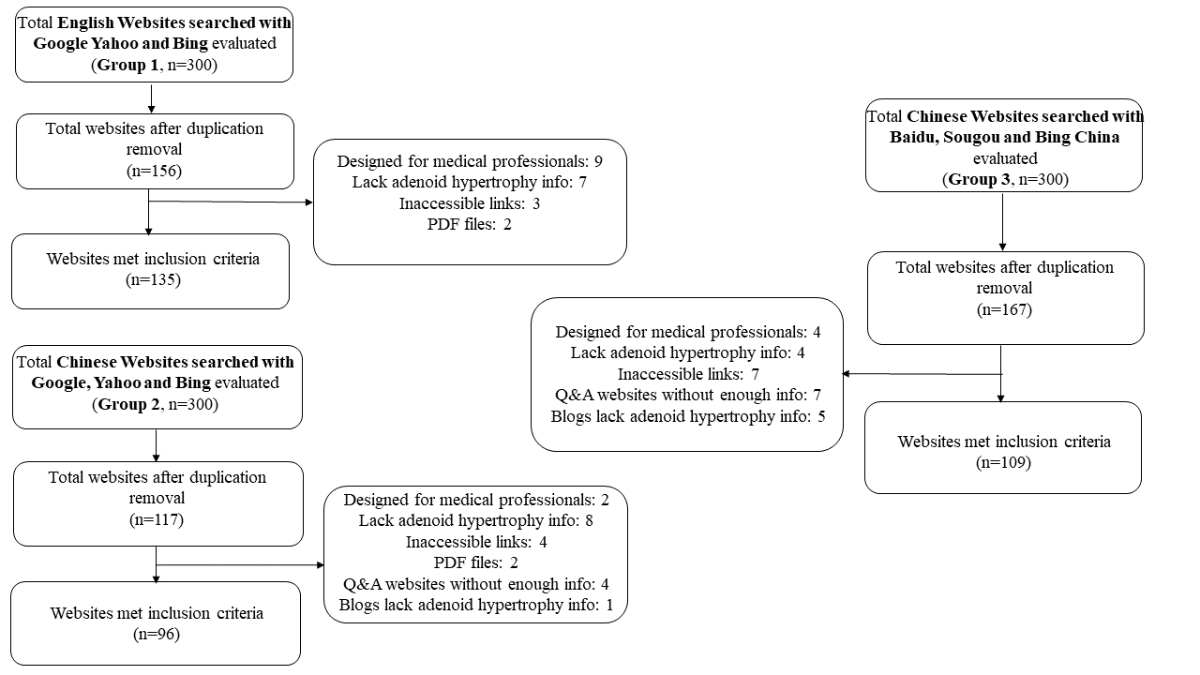
Flow diagram of website inclusion and exclusion. Group 1: English websites searched with Google, Yahoo, and Bing; Group 2: Chinese websites searched with Google, Yahoo, and Bing; Group 3: Chinese websites searched with Baidu, Sougou, and Bing China. Q&A: question and answer.

A total of 340 websites met the inclusion criteria in the three groups; the reasons for exclusion are shown in the flow diagram in Figure 1. Group 1 (English websites searched with Google, Yahoo, and Bing) had 135 websites, group 2 (Chinese websites searched with Google, Yahoo, and Bing) had 96 websites, and group 3 (Chinese websites searched with Baidu, Sougou, and Bing China) had 109 websites. As shown in Figure 1, when searching for the Chinese websites, the chance of visiting a useless website was much higher than when searching English websites, especially when using the Chinese domestic search engines (Baidu, Sougou, or Bing China); 34.73% of websites were excluded from group 3 and 17.95% were excluded from group 2, whereas only 13.46% of websites were excluded in the search of English websites.

**Figure 2:**
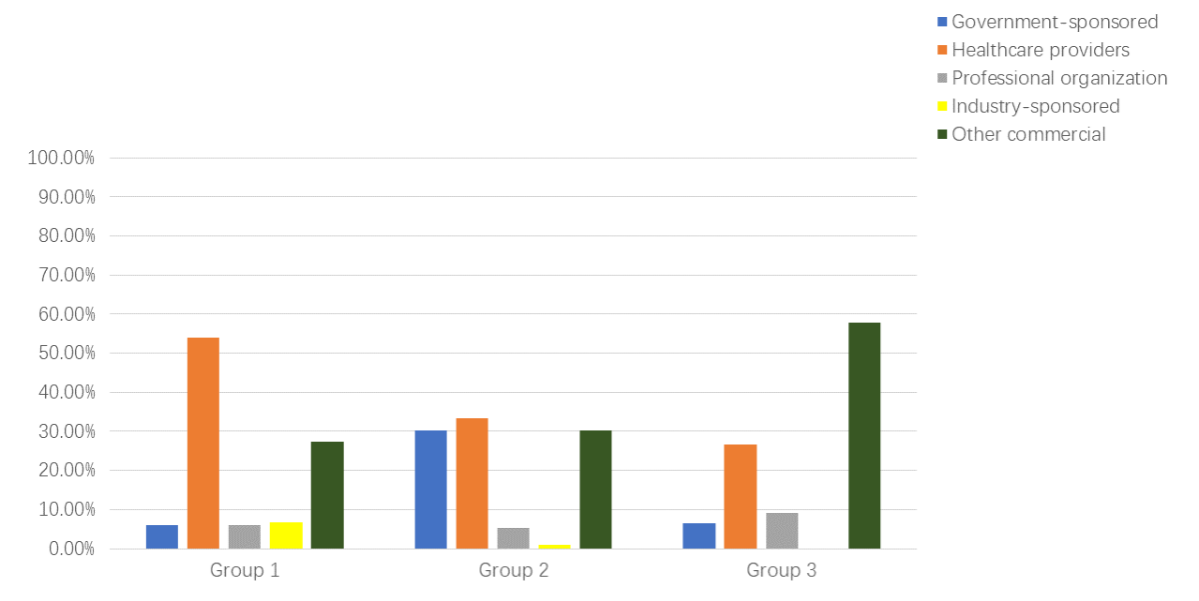
Composition of website types in three different groups. Group 1: English websites searched with Google, Yahoo, and Bing; Group 2: Chinese websites searched with Google, Yahoo, and Bing; Group 3: Chinese websites searched with Baidu, Sougou, and Bing China.

The types of websites in each group are presented in Figure 2. The dominant type in group 1 was health care provider websites (54.1%), followed by other commercial websites (27.4%). The dominant type in group 2 was also health care provider websites (33.3%), followed by government-sponsored websites (30.2%) and other commercial websites (30.2%). However, in group 3, the dominant type was other commercial websites (57.8%), followed by health care provider websites (26.6%). The composition of types of websites was very different among the three groups, especially between the two Chinese search groups (group 2 and group 3). We found several government-sponsored websites (30.2%) when using the Google, Yahoo, and Bing search engines, which dropped to only 6.4% when using the Baidu, Sougou, and Bing China search engines. Moreover, the commercial websites accounted for 57.8% of the Baidu, Sougou, and Bing China group, which was much higher than that of the Google, Yahoo, and Bing group (30.2%).

### Mean Quality Score

**Figure 3:**
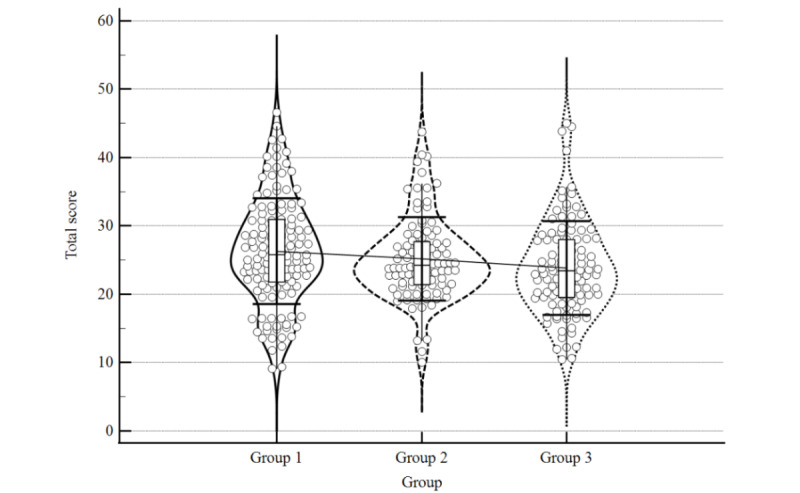
Violin distribution plot of mean total score.

**Figure 4:**
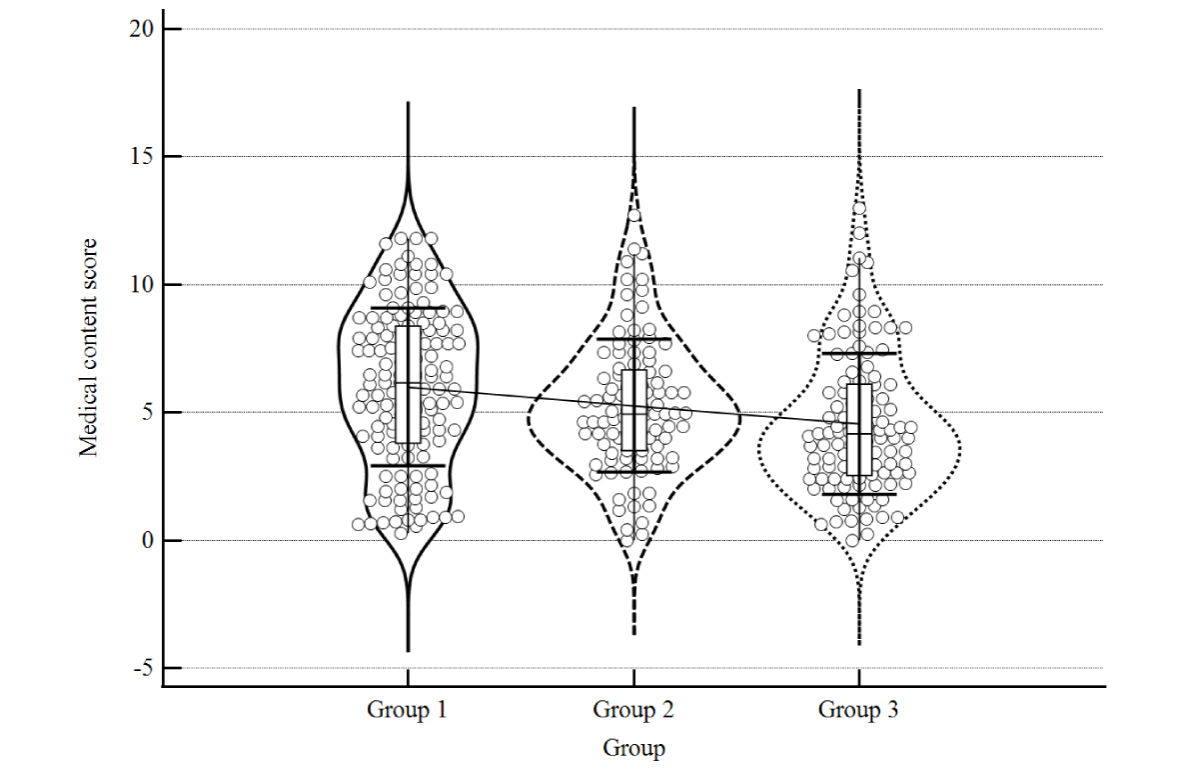
Violin distribution plot of mean medical content score.

**Figure 5:**
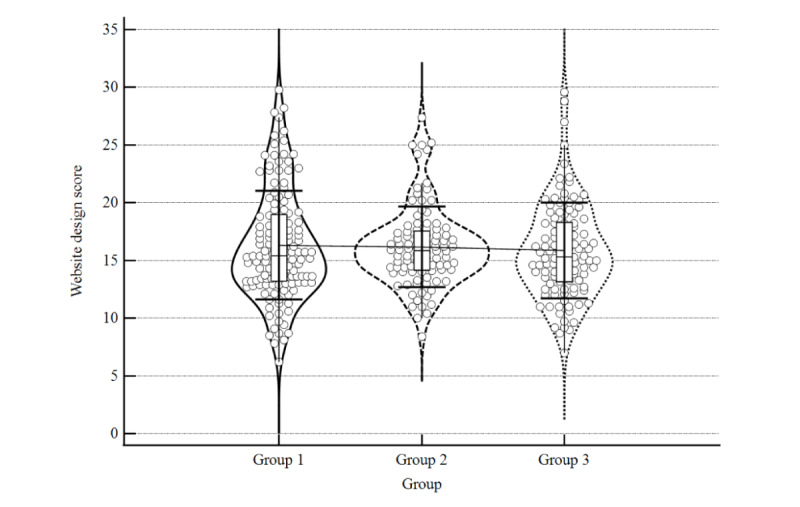
Violin distribution plot of mean website design score.

The individual mean scores for all assessment items are provided in Table S1 of Multimedia Appendix 2, the mean quality scores of each group and subgroup are provided in Table 1, and the distribution of the data is presented with violin plots in Figures 3-5. The distribution plots for individual items are provided in Multimedia Appendix 3. Group 1 had the highest mean total quality score. Group 1 also scored higher for medical content quality than the other two groups. The mean score of website design did not vary substantially among groups. In the subgroups of website types, professional organization websites in group 1 scored the highest both within and between groups, followed by government-affiliated websites in group 1. Among Chinese websites (groups 2 and 3), professional organization websites in group 2 scored the highest for both the total score and medical content score.

**Table 1:** Summary scores for website groups and subgroups.

Website types		Websites, n	Medical content, mean (SD)	Website design, mean (SD)	Total score, mean (SD)
**Group 1 (English websites searched with Google, Yahoo, and Bing)**					
	Government	8	8.18 (2.61)	19.81 (4.92)	33.00 (7.27)
	Health care	73	6.39 (2.73)	15.33 (4.13)	25.97 (6.67)
	Professional	8	8.58 (2.36)	21.73 (4.36)	34.95 (6.48)
	Industry	9	4.87 (3.49)	16.08 (3.60)	24.40 (7.54)
	Commercial	37	4.46 (3.11)	16.37 (5.11)	24.10 (8.43)
	All	135	5.99 (3.08)	16.31 (4.71)	26.30 (7.75)
**Group 2 (Chinese websites searched with Google, Yahoo, and Bing)**					
	Government	29	5.43 (2.85)	16.21 (3.40)	25.33 (6.30)
	Health care	32	5.21 (2.39)	16.61 (3.77)	25.45 (6.18)
	Professional	5	7.60 (4.06)	16.72 (3.52)	28.72 (7.75)
	Industry	1	N/A^a^	N/A	N/A
	Commercial	29	4.76 (2.18)	15.53 (3.36)	24.13 (5.60)
	All	96	5.25 (2.60)	16.16 (3.48)	25.16 (6.08)
**Group 3 (Chinese websites searched with Baidu, Sougou, and Bing China)**					
	Government	7	3.40 (1.39)	16.14 (2.02)	22.68 (4.22)
	Health care	29	4.60 (2.85)	16.05 (4.34)	24.06 (7.07)
	Professional	10	5.32 (3.03)	15.44 (3.47)	24.36 (6.20)
	Industry	0	N/A	N/A	N/A
	Commercial	63	4.54 (2.76)	15.78 (4.38)	23.80 (7.24)
	All	109	4.55 (2.74)	15.84 (4.14)	23.85 (6.88)

^a^N/A: not applicable.

### Cross-Comparisons Among the Three Groups

The Mann-Whitney *U* test (Table 2) showed that the mean total quality score of group 1 was significantly higher than that of group 3 (25.80 vs 23.44, *P*=.005), whereas there was no significant difference between the total scores of groups 1 and 2 (*P*=.13) or groups 2 and 3 (*P*=.09).

In terms of the medical content quality, the significance of cross-comparison was more prominent. Group 1 had a higher mean medical content score than group 2 (6.16 vs 4.94, *P*=.03) and group 3 (6.16 vs 4.16, *P*<.001). Group 2 also had a higher mean medical content score than group 3 (4.94 vs 4.16, *P*=.02). There were no significant differences in the website design score in cross-comparisons between each group.

These results suggested that English websites generally have better-quality medical content than Chinese websites, and that the Chinese websites searched with foreign search engines are of better quality with respect to medical content than websites searched with Chinese domestic search engines.

**Table 2:** Between-group comparisons.

Cross-comparison			Medical content		Website design		Total score
**Group 1 versus group 3: English websites (GYB^a^)** **versus Chinese websites (BSB^b^)**							
	Mann-Whitney *U*	5250.000		7101.000		5812.500	
	*P* value (2-tailed)	<.001		.64		.005	
**Group 2 versus group 3: Chinese websites (GYB) versus Chinese websites (BSB)**							
	Mann-Whitney *U*	4259.000		4915.000		4517.500	
	*P* value (2-tailed)	.02		.45		.09	
**Group 1 versus group 2: English websites (GYB) versus Chinese websites (GYB)**							
	Mann-Whitney U	5385.000		6273.500		5716.000	
	*P* value (2-tailed)	.03		.68		.13	

^a^GYB: Google, Yahoo, and Bing search engines.

^b^BSB: Baidu, Sougou, and Bing China search engines.

### Subgroup Comparisons Within and Between Groups

The subgroup analysis results are presented in Tables S2 and S3 of Multimedia Appendix 2. Within-group analysis for group 1 (Table S2 of Multimedia Appendix 2) showed higher mean total scores for government-sponsored websites and professional organization websites than the other website types (*P*<.05), which was consistent when considering scores for website design and medical content. In group 2, no significance was found in within-group analysis. In group 3, government-sponsored and professional organization websites had higher mean scores than the other three website types (*P*<.05), and government-affiliated websites also scored significantly higher than professional organization websites (*P*<.05).

Significant findings were also found in the between-group analysis (Table S3 in Multimedia Appendix 2). For government-sponsored websites, group 1 had a higher mean total score and medical content score than groups 2 and 3 (*P*<.05). For health care provider websites, group 1 scored the highest in mean medical content (*P*<.05) and group 2 also had a significantly higher mean medical content score than group 3 (*P*<.05). Similarly, for professional organization websites, group 1 had the highest mean total score and website design score (*P*<.05), and group 2 had a higher mean total score than group 3 (*P*<.05). For other commercial websites, there was no difference between groups in either website design or medical content.

### Relationship Between Website Traffic and Quality

We only found weak positive correlations between website traffic and website design score in two groups (Table 3). The Pearson *r* value was 0.177 (*P*=.04) in group 1 and was 0.303 (*P*=.003) in group 2, which suggested that websites with higher traffic generally have a slightly better website design, but the medical content is not necessarily of better quality.

**Table 3:** Correlation between website traffic and quality score.

Group			Website traffic		Medical content score		Website design score		Total quality score
**Group 1 (n=135)**									
	*r* value	1		0.092		0.177		0.156	
	*P* value (2-tailed)	—^a^		.29		.04		.07	
**Group 2 (n=96)**									
	*r* value	1		0.020		0.303		0.177	
	*P* value (2-tailed)	—		.85		.003		.08	
**Group 3 (n=109)**									
	*r* value	1		–0.032		0.019		–0.034	
	*P* value (2-tailed)	—		.74		.85		.73	

^a^Not applicable.

## Discussion

### Group Cross-Comparison

Our study revealed a general phenomenon that English-language users are more likely to have access to higher-quality online medical content than Chinese-language users. It is astonishing to find that Chinese users outside mainland China are more likely to have access to higher-quality Chinese websites when using the Google, Yahoo, and Bing search engines, and they are less likely to come across uninformative websites. The composition of website types was found to be very different between the two Chinese website groups; when using the Google, Yahoo, and Bing search engines, health care provider websites (33.3%) and government-sponsored websites (30.2%) were more often reached, which certainly are of better overall quality according to our subgroup analysis. When using Baidu, Sougou, and Bing China search engines, commercial websites (57.8%) and health care provider websites (26.60%) occurred more often in the search results. Furthermore, the composition of health care provider websites also varied between the two groups. In the Google, Yahoo, and Bing group, 50% of the health care provider websites belong to well-known public hospitals, whereas in the Baidu, Sougou, and Bing China group, all health care provider websites are either private clinics or online consultation platforms, with no public hospital websites included. It is surprising that we barely obtained hits from any government-sponsored websites when using domestic Chinese search engines (Baidu, Sougou, Bing China), despite the fact that the quality of government-sponsored websites is generally higher than that of other types of websites according to our data. Further research on this phenomenon was performed, showing that the monopolistic search engine Baidu (accounting for 65.81% of the market share [14]) has its own SEO service, which can help any websites rank higher on the search result list (not as an advertisement) [18,19]. In other words, without paying for the service, a website is less likely to be seen by the audience regardless of the website’s quality. This can partly explain why we were not able to find public hospital or government-sponsored websites when using the Baidu, Sougou, and Bing China search engines since they never pay to promote themselves. Such commercial manipulation can also explain why only 40.37% of Chinese websites searched with Baidu, Sougou, and Bing China scored in the objectivity item compared to 56.25% and 68.15% of Chinese and English websites, respectively, searched with Google, Yahoo, and Bing. This is because private clinics that paid for SEO are trying to maximize their profit through promoting their products and services, and thus they are less objective.

### Subgroup Analysis

Interesting findings were obtained in our subgroup analysis. In the English website group, government-sponsored and professional organization websites scored the highest in both website design and medical content. This was expected because most of these websites are well-known webpages such as the NCBI library, National Health Service, or different otorhinolaryngologist associations. A similar result was observed in group 3 (Chinese websites searched with Baidu, Sougou, and Bing China), but only with respect to medical content. It is also interesting that in group 2 (Chinese websites searched with Google, Yahoo, and Bing), although the government-sponsored websites accounted for 30.2%, they did not show any superiority in quality over other types of websites. After running through the list of these government websites, it was found that most of them are only from local governments (eg, city, county, district); thus, the lack of medical professionals’ support can likely explain why they did not outcompete the other website types.

### Website Traffic

Weak correlations between website traffic and website design quality were found when using Google, Yahoo, and Bing as the search engines for both English and Chinese websites, whereas no correlation was found in terms of medical content. This indicated that the more widely viewed websites are more likely to have a better overall website design, but that the medical content is not necessarily of good quality. No correlation was found when using Baidu, Sougou, and Bing China as the search engines, which was not surprising due to different searching logic and business strategies.

However, there are limitations when using SEMrush or SEO tools in estimating website traffic. First, their traffic estimates are based on a variety of factors, including keyword search volume and rankings; thus, they can be inaccurate and should be treated as estimates rather than exact figures. Second, these tools only track data for websites that are indexed within the tool, which means that the traffic of all websites may not be captured, especially for newer or less well-known sites. Third, the traffic estimates can fluctuate based on seasonal trends such as increased traffic during holiday periods, which may not be representative of overall website traffic. Finally, SEMrush may not be able to accurately estimate traffic if a website’s traffic sources change over time, such as if more traffic is coming from social media or direct referrals rather than search engines.

### Medical Content Accuracy

In terms of the overall accuracy of medical information, in 14.81% of the websites in the English group, >50% of the information included was considered to be inaccurate and inconsistent with guidelines. This finding is consistent with previous studies on varicose veins (17%) and melanoma (14%) [15,20]. The most inaccurate websites offered homeopathic or herbal therapy, which are not recommended by any official guidelines. In group 2 and group 3, 11.46% and 14.68% of the websites contained >50% inaccurate information, respectively. Although the percentage of websites with >50% inaccurate information in these two Chinese website groups was slightly less than that of the English website group, the English website group still achieved the highest mean accuracy score. This can be attributed to the mention of traditional Chinese medicine (TCM) therapies by many Chinese websites. Although there is evidence that TCM therapies can be of clinical benefit to patients [21], they have not yet been adopted by any official guideline, leading to a classification of inaccurate information based on our analysis method.

### Comparison With Related Studies

A similar study on dementia conducted by Tsiang and Woo [22] revealed that Chinese websites were more likely to be commercial and generally had lower quality than English websites, which is consistent with our findings. However, their research was conducted using Google only, which is not very representative to most Chinese users since approximately 1.4 billion Chinese users do not have access to the Google search engine. Li et al [23] conducted a web search on breast cancer with Baidu and concluded that among all types of Chinese websites, nonprofit websites were more likely to have a higher DISCERN score [24], comprising 16 items assessing a website’s reliability and quality of information. These findings are also very consistent with our results searched with the Baidu, Sougou, and Bing China search engines.

Although these previous studies [22,23] were inspiring and informative, they are not sufficiently comprehensive and representative to make an effective comparison between Chinese and English websites. The Internet Censorship System is likely to create a completely different internet environment in mainland China; thus, when searching and evaluating Chinese websites, the Chinese mainland must be discussed separately. Accordingly, to make our study more inclusive and representative, we conducted the search of English and Chinese websites using the top three search engines worldwide, which can represent English and Chinese users residing outside mainland China, and we also searched Chinese websites using the top three search engines used in mainland China, representing users residing on the Chinese mainland.

### Study Limitations

Several limitations of this study should be acknowledged. First, since the information on webpages is dynamic, in less than 1 month, 14 website links became inaccessible, which makes this type of study extremely time-sensitive and may soon become outdated. Second, we did not compare the readability between Chinese and English websites since there is no clear method for comparing readability between different languages. Third, this study did not consider geographic variation based on IP locations. Moreover, since some items of website assessment were evaluated subjectively, the scoring process can potentially be influenced by different factors that could lead to implicit or cognitive bias. Finally, we did not consult the Health On the Net (HON) certification code, which limited the reliability of the quality assessment of the included websites.

### Conclusions

This study is the first to analyze and compare the quality of online medical information between Chinese and English websites while taking the Internet Censorship System into consideration, which can contribute to gaining a better understanding of whether the language barrier is stopping people from obtaining accurate health information and if internet isolation is hindering people’s access to better-quality online medical information. The results demonstrate that the Internet Censorship System is hindering access to more accurate health information for Chinese mainland users. Government-sponsored and professional organization websites exhibited superiority in medical information accuracy over other types of websites both in the English and Chinese (mainland) groups, indicating that these types of websites deserve higher ranks on the search list when seeking medical information on the internet. Future work is warranted to make websites with good-quality medical information more readily available to general users by cooperating with search engine companies and efficiently promoting high-quality medical websites.

## Appendix

Multimedia Appendix 1 Tool design and the data collecting sheet.

Multimedia Appendix 2 Tables S1-S3.

Multimedia Appendix 3 Violin plots for each item in the scoring scale.

## Abbreviations

HON: Health On the Net
NCBI: National Center for Biotechnology Information
SAR: Special Administrative Region
SEO: search engine optimization
TCM: traditional Chinese medicine

## Data Availability

All of the included websites are open access and can be accessed through browsers; the included websites and objective scoring system are provided in Multimedia Appendices 1-3.
