# Supplementary material for: Critical Analysis and Cross-Comparison Between English and Chinese Websites Providing Online Medical Information for Patients With Adenoid Hypertrophy: Cross-sectional Study
Source: JMIR Form Res. 2023 Apr 24;7:e44010. doi: 10.2196/44010 (PMC10167579; doi:10.2196/44010)
Supplement: Multimedia Appendix 2 [file formative_v7i1e44010_app2.docx]

**Table S1.** Individual mean scores in each item.

|  | Group 1 | Group 2 | Group 3 |
| --- | --- | --- | --- |
|  |  |  |  |
| **Accessibility (total 1 score)** |  |  |  |
| Does the link lead directly to information on adenoid hypertrophy? | 0.81±0.39 | 0.94±0.24 | 1.00±0.00 |
| **Accountability (total 15 scores)** |  |  |  |
| Is the author clearly identified? | 0.35±0.48 | 0.64±0.48 | 0.64±0.48 |
| Is the author's affiliation clearly identified? | 0.23±0.42 | 0.49±0.50 | 0.53±0.50 |
| Are the authors' credentials clearly indicated? | 0.17±0.38 | 0.28±0.45 | 0.50±0.50 |
| Are sources of published information cited at the end or in the body of the document? | 0.22±0.42 | 0.08±0.28 | 0.09±0.29 |
| Is there a range of sources of information used to compile the website? [3 or more sources cited(1 score); Less than 3 sources cited (0 score)] | 0.13±0.34 | 0.03±0.17 | 0.04±0.19 |
| Are the resources used to compile the website reliable? (e.g., Do the authors use journal articles, peer-reviewed sites like UpToDate or e-Medicine, academic or government sites, textbooks). [2 or more reliable sources used (2 scores); 1 reliable source used (1 score); No reliable sources used (0 score)] | 0.39±0.76 | 0.10±0.42 | 0.12±0.42 |
| Are ownership of the site disclosed? | 0.87±0.34 | 0.90±0.31 | 0.78±0.41 |
| Is the date of creation or date of modification specified? | 0.45±0.50 | 0.85±0.35 | 0.78±0.41 |
| How current is the last update to the site (from date site is being assessed)? [Less than 2 years ago(2 scores); Between 2 and 4 years ago(1 score)] Over 4 years ago or unknown (0) | 0.53±0.78 | 0.76±0.93 | 0.10±0.43 |
| How many external links are provided? [2 or more links provided (2 scores); 1 link provided (1 score); No links provided (0 score)] | 0.13±0.41 | 0.04±0.29 | 0.15±0.52 |
| Are the external links (not advertising) provided functional (check up to five links in the order that they appear)? [50% or greater of links are accessible (2 scores); Less than 50% of links are accessible or if only 1 link available (1 score); No links provided or none of them are accessible (0)] | 0.13±0.41 | 0.04±0.29 | 0.17±0.55 |
| SUM of accountability (total 15 scores) | 3.59±3.03 | 4.22±2.13 | 3.90±2.19 |
| **Interactivity (total 5 scores)** |  |  |  |
| Does the site offer a within-site search engine? | 0.75±0.43 | 0.73±0.44 | 0.65±0.48 |
| Does the site offer audio or video support? | 0.14±0.35 | 0.15±0.35 | 0.27±0.44 |
| Does the site offer patient discussion boards or forums? | 0.06±0.24 | 0.29±0.45 | 0.54±0.50 |
| Does the site provide contact information for the author or webmaster with respect to information on the site? | 0.07±0.26 | 0.24±0.43 | 0.35±0.48 |
| Does the site offer educational support (e.g., workshops, modules, evaluation surveys)? | 0.03±0.17 | 0.00±0.00 | 0.00±0.00 |
| SUM of interactivity (total 5 scores) | 1.05±0.70 | 1.41±0.84 | 1.81±0.98 |
| **Structure and organization (total 5 scores)** |  |  |  |
| Is heading used to structure the text? | 1.00±0.00 | 0.99±0.10 | 1.00±0.00 |
| Is subheading used to structure the text? | 0.98±0.15 | 0.68±0.47 | 0.74±0.44 |
| Does the website contain diagrams/table/pictures? | 0.57±0.50 | 0.37±0.48 | 0.50±0.50 |
| Does website contain hyperlink within the body of the text? | 0.57±0.50 | 0.07±0.26 | 0.14±0.34 |
| Is advertisement absent? | 0.77±0.42 | 0.68±0.47 | 0.48±0.50 |
| SUM of structure and organization (total 5 scores) | 3.89±0.81 | 2.78±1.01 | 2.86±1.12 |
| **Accuracy (total 5 scores)** |  |  |  |
| Is there conflicting information provided when compared to other reliable sources? Completely inaccurate (0) Less than 25% accurate (1) 25-50% accurate (2) 50-75% accurate (3) >75% accurate (4) 100% accurate (5) | 3.99±1.24 | 3.75±1.03 | 3.45±0.90 |
| **Content (total 15 scores)** |  |  |  |
| Is adenoid hypertrophy accurately defined? | 0.33±0.47 | 0.50±0.50 | 0.44±0.50 |
| Incidence and prevalence of adenoid hypertrophy defined? | 0.10±0.29 | 0.14±0.37 | 0.14±0.34 |
| Etiology of adenoidal hypertrophy explained? | 0.90±0.29 | 0.78±0.41 | 0.73±0.44 |
| How is adenoid hypertrophy prevented? | 0.06±0.24 | 0.31±0.46 | 0.33±0.47 |
| How many symptoms mentioned in the article? 0.1 point for each symptom, maximal 1 point | 0.70±0.27 | 0.74±0.33 | 0.65±0.34 |
| When patients should seek medical attention provided? | 0.26±0.44 | 0.34±0.47 | 0.47±0.50 |
| Diagnostic test related to adenoidal hypertrophy described? | 0.50±0.50 | 0.33±0.47 | 0.31±0.46 |
| Conservative management of adenoidal hypertrophy provided? | 0.77±0.42 | 0.74±0.44 | 0.55±0.50 |
| How many kinds of medication treatment were mentioned in the article?（>=2 kinds get 1 point, 1 kind get 0.5 point) | 0.55±0.41 | 0.30±0.44 | 0.31±0.50 |
| Selection of surgical indication provided? | 0.75±0.43 | 0.58±0.49 | 0.60±0.49 |
| Procedures /Surgeries for tonsil and adenoidal hypertrophy described? Procedure defined (0.2)  Procedure process described (0.2)  Recovery/postoperative care explained (0.2) Complication explained(0.2) Surgical method (0.2) | 0.41±0.27 | 0.30±0.23 | 0.28±0.29 |
| Did the website mentioned the tonsillectomy or adenotonsillectomy? | 0.56±0.50 | 0.26±0.44 | 0.28±0.45 |
| Prognosis of adenoidal hypertrophy (1 point for each: complication of adenoidal hypertrophy described; effectiveness of treatment described) Both (2) Only one present (1) Neither (0) | 0.53±0.63 | 0.99±0.57 | 0.95±0.58 |
| Is there no bias or opinion expressed when a writer interprets or analyzes facts? (e.g., Is the use of viewpoints or persuasive language absent?) | 0.68±0.47 | 0.56±0.50 | 0.40±0.49 |
| SUM of contents (total 15 scores) | 7.09±2.57 | 6.86±2.55 | 6.44±3.20 |

**Table S2. Within-group post hoc SNK-q analysis for subgroups**

| **Group** | **Subgroup** | **Group 1** | | | **Group 2** | | | **Group 3** | | |
| --- | --- | --- | --- | --- | --- | --- | --- | --- | --- | --- |
|  |  |  | **Alpha = 0.05** | |  | **Alpha = 0.05** | |  | **Alpha = 0.05** | |
|  |  | **N** | **1** | **2** | **N** | **1** | **2** | **N** | **1** | **2** |
| **Medical content** | **Commercial** | 37 | 4.46 |  | 29 | 4.76 |  | 63 | 4.52 |  |
|  | **Industry** | 9 | 4.87 |  | - | - | - | - | - | - |
|  | **Healthcare** | 73 | 6.39 | 6.39 | 32 | 5.14 |  | 29 | 3.80 |  |
|  | **Gov** | 8 |  | 8.18 | 29 | 5.47 |  | 7 |  | 7.04 |
|  | **Professional** | 8 |  | 8.58 | 5 | 7.21 |  | 10 | 5.19 | 5.19 |
|  | **Sig.** |  | .22 | .14 |  | .08 |  |  | .37 | .07 |
| **Website design** | **Healthcare** | 73 | 15.33 |  | 32 | 15.53 |  | 29 | 15.64 |  |
|  | **Industry** | 9 | 16.08 |  | - | - | - | - | - | - |
|  | **Commercial** | 37 | 16.37 |  | 29 | 16.69 |  | 63 | 15.73 |  |
|  | **Gov** | 8 | 19.81 | 19.81 | 29 | 15.91 |  | 7 | 16.54 |  |
|  | **Professional** | 8 |  | 21.73 | 5 | 18.50 |  | 10 | 16.69 |  |
|  | **Sig.** |  | .06 | .28 |  | .13 |  |  | .92 |  |
| **Total score** | **Commercial** | 37 | 24.10 |  | 29 | 24.87 |  | 63 | 23.51 |  |
|  | **Industry** | 9 | 24.40 |  | - | - | - | 7 | 27.15 |  |
|  | **Healthcare** | 73 | 25.97 |  | 32 | 24.52 |  | 29 | 23.10 |  |
|  | **Gov** | 8 |  | 33.00 | 29 | 25.31 |  | 7 | 27.15 |  |
|  | **Professional** | 8 |  | 34.95 | 5 | 29.71 |  | 10 | 25.88 |  |
|  | **Sig.** |  | .80 | .51 |  | .13 |  |  | .42 |  |

**Table S3. Between-group post hoc SNK-q analysis for subgroups**

|  | **Group** | **Government-sponsored** | | | **Healthcare providers** | | | **Professional organization** | | |
| --- | --- | --- | --- | --- | --- | --- | --- | --- | --- | --- |
|  |  |  | **Alpha = 0.05** | |  | **Alpha = 0.05** | |  | **Alpha = 0.05** | |
|  |  | **N** | **1** | **2** | **N** | **1** | **2** | **N** | **1** | **2** |
| **Medical content** | **1** | 8 |  | 8.18 | 73 |  | 6.39 | 8 | 8.58 |  |
|  | **2** | 29 | 5.43 |  | 32 | 5.21 | 5.21 | 5 | 7.60 |  |
|  | **3** | 7 | 3.40 |  | 29 | 4.60 |  | 10 | 5.32 |  |
|  | **Sig.** |  | .10 | 1.00 |  | .32 | .06 |  | .14 |  |
| **Website design** | **1** | 8 | 19.81 |  | 73 | 15.33 |  | 8 |  | 21.73 |
|  | **2** | 29 | 16.21 |  | 32 | 16.61 |  | 5 | 16.72 |  |
|  | **3** | 7 | 16.14 |  | 29 | 16.05 |  | 10 | 15.44 |  |
|  | **Sig.** |  | .07 |  |  | .37 |  |  | .54 | 1.00 |
| **Total score** | **1** | 7 | 22.68 |  | 73 | 25.97 |  | 8 |  | 34.95 |
|  | **2** | 29 | 25.33 |  | 32 | 25.45 |  | 5 | 28.72 | 28.72 |
|  | **3** | 8 |  | 33.00 | 29 | 24.06 |  | 10 | 24.36 |  |
|  | **Sig.** |  | .35 | 1.00 |  | .43 |  |  | .23 | .09 |
